# A retrospective descriptive analysis of non-physician-performed prehospital endotracheal intubation practices and performance in South Africa

**DOI:** 10.1186/s12873-022-00688-4

**Published:** 2022-07-16

**Authors:** Craig A. Wylie, Farzana Araie, Clint Hendrikse, Jan Burke, Ivan Joubert, Anneli Hardy, Willem Stassen

**Affiliations:** 1 Division of Emergency Medicine, University of Cape Town, Cape Town, South Africa; 2 Department of Anaesthesia and Perioperative Medicine, University of Cape Town, Cape Town, South Africa

**Keywords:** Prehospital emergency care, Airway management, Endotracheal intubation, South Africa

## Abstract

**Introduction:**

Prehospital advanced airway management, including endotracheal intubation (ETI), is one of the most commonly performed advanced life support skills. In South Africa, prehospital ETI is performed by non-physician prehospital providers. This practice has recently come under scrutiny due to lower first pass (FPS) and overall success rates, a high incidence of adverse events (AEs), and limited evidence regarding the impact of ETI on mortality. The aim of this study was to describe non-physician ETI in a South African national sample in terms of patient demographics, indications for intubation, means of intubation and success rates. A secondary aim was to determine what factors were predictive of first pass success.

**Methods:**

This study was a retrospective chart review of prehospital ETIs performed by non-physician prehospital providers, between 01 January 2017 and 31 December 2017. Two national private Emergency Medical Services (EMS) and one provincial public EMS were sampled. Data were analysed descriptively and summarised. Logistic regression was performed to evaluate factors that affect the likelihood of FPS.

**Results:**

A total of 926 cases were included. The majority of cases were adults (*n* = 781, 84.3%) and male (*n* = 553, 57.6%). The most common pathologies requiring emergency treatment were head injury, including traumatic brain injury (*n* = 328, 35.4%), followed by cardiac arrest (*n* = 204, 22.0%). The mean time on scene was 46 minutes (SD = 28.3). The most cited indication for intubation was decreased level of consciousness (*n* = 515, 55.6%), followed by cardiac arrest (*n* = 242, 26.9%) and ineffective ventilation (*n* = 96, 10.4%). Rapid sequence intubation (RSI, *n* = 344, 37.2%) was the most common approach. The FPS rate was 75.3%, with an overall success rate of 95.7%. Intubation failed in 33 (3.6%) patients. The need for ventilation was inversely associated with FPS (OR = 0.42, 95% CI: 0.20–0.88, *p* = 0.02); while deep sedation (OR = 0.56, 95% CI: 0.36–0.88, *p* = 0.13) and no drugs (OR = 0.47, 95% CI: 0.25–0.90, *p* = 0.02) compared to RSI was less likely to result in FPS. Increased scene time (OR = 0.99, 95% CI: 0.985–0.997, *p* < 0.01) was inversely associated FPS.

**Conclusion:**

This is one of the first and largest studies evaluating prehospital ETI in Africa. In this sample of ground-based EMS non-physician ETI, we found success rates similar to those reported in the literature. More research is needed to determine AE rates and the impact of ETI on patient outcome. There is an urgent need to standardise prehospital ETI reporting in South Africa to facilitate future research.

**Supplementary Information:**

The online version contains supplementary material available at 10.1186/s12873-022-00688-4.

## Introduction

Prehospital advanced airway management is one of the most commonly performed invasive interventions in the out-of-hospital setting [1–3]. Protecting the airway of a critically ill or injured patient and facilitating adequate ventilation and oxygenation is an essential part of prehospital emergency care [2]. The skill of endotracheal intubation (ETI) is normally reserved for only the highest qualified prehospital providers and, depending on the prehospital system and available resources, often only to anaesthetists or emergency physicians who practice in the prehospital phases of care [2]. Owing to conflicting results on the safety and impact on mortality of prehospital ETI performed by non-physicians, this practice has come under immense scrutiny in recent times [2].

Controversies surrounding non-physician performed ETI relate mostly to lower first pass (FPS) and overall success rates [4], or poorer outcome associated with prehospital ETI, especially in traumatic brain injury [5, 6]. A more recent systematic review and meta-analysis found that a marginal difference in the overall ETI rates between physicians (99%) and non-physicians (97%) and a 10% difference in first pass intubation success when comparing physicians (88%) versus non-physicians (78%). Fouche et al. also reported a higher rate of adverse events (AEs) among non-physicians, which may be explained by a lower FPS rate in this cohort [2]. Almost all cited studies originate from a higher income country (HIC) setting.

There are important differences in prehospital and emergency care systems in low-to middle-income countries (LMICs) that data originating from HICs do not take into consideration. Firstly, prehospital services in LMICs are predominantly non-physician based [7] because of a critical shortage of physicians [8]. Secondly, LMICs may have significantly prolonged prehospital times because of proximity to hospital [9]. Patients also experience many barriers to accessing emergency care [10], delaying presentation. Lastly, LMICs suffer from unique burdens of disease including injury, infectious disease (including human immunodeficiency virus and tuberculosis) and chronic non-communicable diseases [9]. All these factors may make the need for earlier critical interventions in the prehospital setting, including prehospital ETI [11].

South Africa has one of the most developed emergency medical services (EMS) systems on the African continent [7]. Here, prehospital advanced life support non-physicians have been performing prehospital ETI for well over a decade [11, 12]. Yet, there is still a paucity of literature to assess the safety and impact of non-physician performed ETI originating from LMICs, including South Africa. Where literature exists, it frequently originates from a single centre [6, 11], student paramedics [13] or from the aeromedical environment [14]. The aim of this study was to describe non-physician ETI in a South African national sample in terms of patient demographics, indications for intubation, means of intubation and success rates. A secondary aim was to determine what factors are predictive of first pass success.

## Methods

We performed a retrospective chart review of pre-hospital ETIs performed by non-physician prehospital providers, between the periods of 01 January 2017 to 31 December 2017. Two national private EMS and one provincial public EMS were sampled. This manuscript has been prepared in accordance with the The REporting of studies Conducted using Observational Routinely-collected health Data (RECORD) extension of the Strengthening the Reporting of Observational Studies in Epidemiology (STROBE) checklist [15].

### Setting

South Africa is an upper-middle income country with an estimated population of approximately 58 million people. There are two distinct healthcare systems, private healthcare and state healthcare. State healthcare is that provided by the South African government to citizens while private healthcare is only accessible to patients through funds to pay for the services, or those with healthcare insurance aid. Only 17% of South Africans currently belong to a healthcare insurance scheme [16]. In the context of EMS though, private EMS are mandated by the constitution to provide emergency care to all patients regardless of their ability to pay or insurance status. Private EMS are generally better-resourced and have faster response times than provincial EMS [17], but follow the same national guidelines and scopes of practice.

Together, the services included in this study receive approximately 150,000 incoming calls per month. The two private EMS provide national coverage in all provinces, while the provincial EMS sampled in this study provide coverage only to the Western Cape province of South Africa. Approximately 10% of South Africa’s population live in the Western Cape. These services provide care to rural and urban populations.

In South Africa prehospital emergency care is provided by non-physician prehospital care providers. Although many cadres of prehospital providers exist, only advanced life support (ALS) providers may perform endotracheal intubation. These providers, who most often respond on a single crewed rapid response vehicle, may either be qualified through a vocational training (1 year certificate course) or higher education training (three-year university diploma or four-year university honours degree). While this changed in 2020, during the study period, certificate and diplomat prehospital providers were able to intubate only via deep sedation (or no sedation) while degree holders were licensed to perform rapid sequence intubation (RSI). RSI is performed with a choice of ketamine or etomidate for induction and succinylcholine or rocuronium for neuromuscular blockade. Deep sedation-only ETI is performed with either midazolam alone or a combination of midazolam and morphine. No sedation ETI is generally indicated in instances of cardiac arrest or where a patient is deeply unconscious without a gag reflex. After 2020, endotracheal intubation of any form is reserved for degree paramedics only [18, 19].

### Sample and sampling

Instances of ETI were identified in a variety of ways, depending on the type of the patient report form or archiving systems of each EMS. For the first national private EMS, hand-written, scanned patient report forms (PRFs) of all patients who were intubated by non-physician prehospital providers, between the periods of 01 January 2017 to 31 December 2017 were eligible for analysis. A standard checkbox on the patient report form indicates that intubation was performed, as well as the number of intubation attempts. Both of these are captured onto a central billing system before the form is scanned for archiving. This allowed for the easy identification of intubated patients.

Both the second national private and provincial public EMS utilise electronic patient report forms (ePRFs). For this reason, an extract of cases that fit our inclusion criteria were extracted. In these cases, the number of intubation attempts is not a field in the ePRF and thus has to be extracted from the narrative, typed clinical notes of the prehospital care provider.

Any patients who were intubated by a physician, those who underwent intubation for interfacility transfer, and those intubated on the aeromedical platforms were excluded from analysis. Patients who were intubated by crew members from another service who were attending to the same scene were also excluded.

### Data extraction and definitions

After specific training in the research aims, objectives, data variables, and the contents of the PRFs, data were extracted according to a dedicated, standard data abstraction form by a data capturer with experience in clinical administration and the authors (FA, JB). Regular meetings between the data capturers and authors were held to ensure credibility of the extraction process. The data extraction form was based on the Utstein reporting guidelines for prehospital advanced airway management [20].

An intubation attempt was defined as the placement of a laryngoscope blade into the pharynx with the aim of exposing the glottis. An intubation success was defined as placement of the distal end of the endotracheal tube and cuff into the patient’s trachea as confirmed by waveform capnography and/or chest auscultation. First Pass Success (FPS) refers to the situation where intubation was successful after a single attempt. If intubation was successful after more than one attempt, this was referred to as Overall Success. A failed intubation was defined as an inability to place an endotracheal tube.

For specific Utstein clinical variables, predicted airway difficulty, and aggravating factors, the PRFs were assessed and interpreted by one of the investigators with clinical experience in anaesthesia and/or prehospital care. If there was a case in which there was uncertainty or dispute, the investigators discussed that case in order to make a joint decision on the variable in question in order to resolve the uncertainty, by consensus.

Lastly, a 10% random sample was drawn for manual verification of accuracy of the data capture. Further manual verification of all discrepant and missing data was undertaken. Where necessary, disputes were resolved by a third investigator.

### Data analysis

Regardless of the data source, data were extracted onto a Microsoft Excel (Microsoft Corporation, Redmond, Washington, United States) spreadsheet. All analyses were conducted using Stata 17.0 (StataCorp, Texas, United States). Continuous variables were summarised as mean and standard deviation; while nominal and ordinal variables were summarised as counts and percentages.

Logistic regression was performed to evaluate the effect of age, sex, reason for emergency treatment, indication for intubation, approach, risk factors, aggravating conditions and scene time on the likelihood of three outcomes: 1) First Pass Success; 2) Overall Success; and 3) Failed Intubation. The outcome was predicted perfectly for two reason variables (infection (including sepsis) and psychiatry (e.g. agitation/psychosis)) and two indication variables (humanitarian and failure of airway device). These variables were thus omitted in the models. This resulted in nine cases being excluded from the FPS model, 83 cases excluded from the overall success model and 220 cases excluded from the failed intubation model.

Model fit was assessed based on the Hosmer-Lemeshow (HL) goodness of fit test and inspection of plots for influential observations. Results from the HL tests indicated reasonable fit for all models. Multicollinearity was assessed using the variance inflation factor (VIF). A VIF is derived for each predictor in the predictor set reflecting the variance by which the estimated coefficient is increased due to near-linear dependences among the predictors. VIFs exceeding 10 indicates that the associated regression coefficients are poorly estimated because of multicollinearity [21]. Cardiac arrest and cardiopulmonary resuscitation (CPR) as indications for intubation both had VIF values greater than 10. Cardiac arrest as an indication was excluded and CPR kept for subsequent models. The indicators for “whether or not aggravating conditions were assessed” and “no aggravating conditions indicated”, also had VIF values larger than 10. Both indicators were kept in the models since it is likely that the distinction between “not assessed” and “no aggravating conditions” cannot be clearly delineated retrospectively.

Cases that were outlying from the other observations in terms of standardised Pearson residuals, leverage values and difference of Chi-square values were excluded from follow-up runs of the models, to evaluate estimates without these observations. Standard errors on the coefficients for the Failed Intubation model improved markedly with the exclusion of one particular case.

## Results

**Fig. 1:**
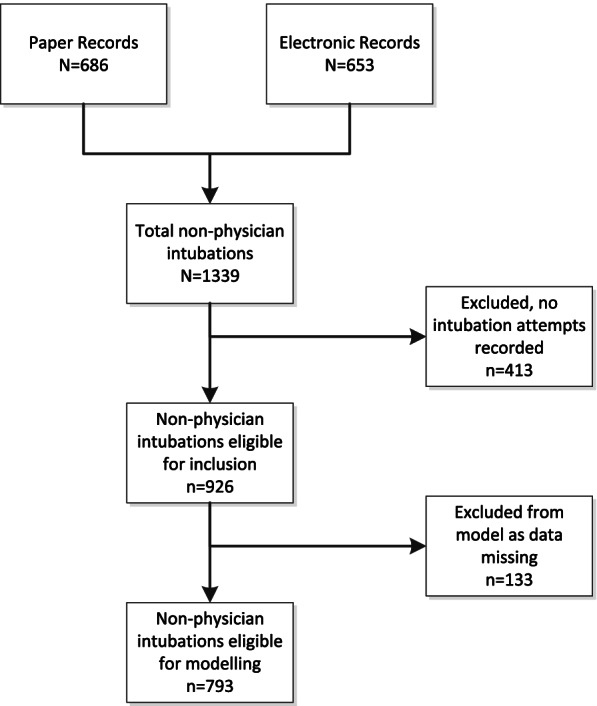
Data sampling and inclusion

A total of 1339 patients received non-physician performed ETI during the study period. The number of intubation attempts were not recorded in 413 (30.8%) patients, and these were therefore excluded from the study as it fell outside our inclusion criteria. Figure 1 outlines the sampling process and exclusion. This yielded a final sample size of 926 cases with 793 (85.6%) cases having complete data and 133 (14.4%) cases with at least one missing data point. These cases were therefore excluded from the regression models.

Table 1 describes the demographics of all available cases. The majority of cases were adults (*n* = 781, 84.3%) and male (*n* = 553, 57.6%). The most common reasons requiring emergency treatment were head injury, including traumatic brain injury (TBI, *n* = 328, 35.4%), followed by cardiac arrest (*n* = 204, 22.0%), and blunt trauma (*n* = 126, 13.6%). The mean time on scene was 46 minutes (SD = 28.3).

**Table 1:** Patient demographics and ETI success

	FPS ***n*** = 697 (%)	Overall success ***n*** = 886 (%)	TOTAL ***n*** = 926 (%)
**Age, n (SD)**			
Child	54 (7.8)	77 (8.7)	79 (8.5)
Adult	597 (85.7)	743 (83.9)	781 (84.3)
Unknown	46 (6.6)	66 (7.5)	66 (7.1)
**Sex (practitioner assigned)**			
Male	391 (56.1)	518 (58.6)	533 (57.6)
Female	288 (41.3)	346 (39.1)	371 (40.1)
Unknown	18 (2.6)	22 (2.5)	22 (2.4)
**Predominant reason for emergency treatment**			
Trauma			
*Head injury, incl. TBI*	243 (34.9)	325 (36.7)	328 (35.4)
*Blunt*	95 (13.6)	115 (13.0)	126 (13.6)
*Penetrating*	24 (3.4)	24 (2.7)	25 (2.7)
*Other*	21 (3.0)	23 (2.6)	23 (2.5)
Medical			
*Cardiac arrest*	153 (22.0)	195 (22.0)	204 (22.0)
*Intoxication*	46 (6.6)	56 (6.3)	62 (6.7)
*Respiratory distress or difficulties*	44 (6.3)	55 (6.2)	58 (6.3)
*Other*	10 (1.4)	13 (1.5)	13 (1.4)
Neurology			
*Stroke*	36 (5.2)	49 (5.5)	54 (5.8)
*Other*	24 (3.4)	30 (3.4)	30 (3.2)

*FPS* First pass success, *TBI* Traumatic brain injury

In Table 2, we present the reasons for intubation as well as the approach taken for intubation. The most cited indication for intubation was decreased level of consciousness (*n* = 515, 55.6%), followed by cardiac arrest (*n* = 242, 26.9%) and ineffective ventilation (*n* = 96, 10.4%). RSI (*n* = 344, 37.2%) was the most common mode of intubation, followed by deep sedation (*n* = 256, 27.7%) and CPR (*n* = 236, 25.5%).

**Table 2:** Indications for and approach to ETI and ETI success

	FPS ***n*** = 697 (%)	Overall success ***n*** = 886 (%)	TOTAL ***n*** = 926 (%)
**Indication for ETI (multiple/patient)**			
Decreased LOC	382 (54.8)	497 (56.1)	515 (55.6)
Cardiac Arrest	192 (27.6)	240 (27.1)	249 (26.9)
Ineffective ventilation	62 (8.9)	85 (9.6)	96 (10.4)
Existing airway obstruction	33 (4.7)	42 (4.7)	49 (5.3)
Combative or uncooperative	42 (6.0)	47 (5.3)	48 (5.2)
Impending airway obstruction	39 (5.6)	41 (4.6)	45 (4.9)
Hypoxemia	7 (1.0)	10 (1.1)	11 (1.2)
Humanitarian (e.g. pain relief)	7 (1.0)	7 (0.8)	7 (0.8)
Failure of A/W device	1 (0.1)	1 (0.1)	1 (0.1)
**Approach**			
RSI	271 (38.9)	337 (38.0)	344 (37.2)
Deep Sedation	179 (25.7)	238 (26.9)	256 (27.7)
Cardiac Arrest	181 (26.0)	226 (25.5)	236 (25.5)
No Medication	65 (9.3)	84 (9.5)	89 (9.6)

*FPS* First pass success, *ETI* Endotracheal intubation, *LOC* Level of consciousness, *A/W* Airway, *RSI* Rapid sequence intubation

Table 3 presents the risk factors for difficult intubation and aggravating conditions for airway management. In the 584 cases where risk factors were assessed, only 68 (11.6%) cases stated that no risk factors were present. Of other cases, *n* = 363 (62.2%) had reduced neck mobility (including manual in-line neck stabilisation), 205 (35.1%) had fluid in the airways, while 72 (12.3%) cases had significant facial or airway trauma reported.

**Table 3:** Risk factors and aggravating conditions and ETI success

	FPS ***n*** = 697 (%)	Overall success ***n*** = 886 (%)	TOTAL ***n*** = 926 (%)
**Patient risk factors for difficult intubation**^**a**^			
Reduced neck mobility (incl. MILNS)	266 (38.2)	355 (40.17)	363 (39.2)
Risk factors not assessed	269 (38.6)	324 (36.6)	342 (36.9)
Fluid in airways	145 (20.8)	200 (22.6)	205 (22.1)
Significant facial or airway trauma	56 (8.0)	70 (7.9)	72 (7.8)
No risk factors for difficult intubation	55 (7.9)	63 (7.1)	68 (7.3)
Severe obesity or thick/short neck	11 (1.6)	18 (2.0)	20 (2.2)
Other	11 (1.6)	15 (1.7)	16 (1.7)
Limited mouth opening	5 (0.7)	11 (1.2)	12 (1.3)
Pre-existing airway device ineffective	6 (0.9)	7 (0.8)	9 (1.0)
Prior difficult intubation	5 (0.7)	7 (0.8)	8 (0.9)
Short TMD	2 (0.3)	3 (0.3)	3 (0.3)
**Aggravating conditions for airway management**^**b**^			
Not assessed	144 (20.7)	174 (19.6)	194 (21.0)
Darkness	68 (9.8)	95 (10.7)	96 (10.4)
In stationary ambulance	42 (6.0)	50 (5.6)	57 (6.2)
Patient entrapped	35 (5.0)	43 (4.9)	45 (4.9)
Hostile environment	35 (5.0)	40 (4.5)	42 (4.5)
In moving ambulance	23 (3.3)	28 (3.2)	29 (3.1)
Not 360-degree access	12 (1.7)	13 (1.5)	15 (1.6)
Bright light/sunlight	9 (1.3)	12 (1.4)	12 (1.3)
Suboptimal provider positioning	4 (0.6)	5 (0.6)	6 (0.7)

*FPS* First pass success, *MILNS* Manual in-line neck stabilisation, *TMD* Thyromental distance

^a^Individual cases may have > 1 risk factor

^b^Some cases had no aggravating condition

In instances where there was a documented assessment of the aggravating conditions for airway management (*n* = 732), 479 (65.4%) cases had no aggravating conditions. Darkness (*n* = 96,13.1%) and intubation in a stationary ambulance (*n* = 57, 7.79%) were the most common aggravating conditions. In 45 (6.2%) cases the patient was entrapped during intubation while there were hostile conditions on scene in 42 (5.8%) cases.

First pass success (FPS) was achieved in 697 patients, yielding an FPS rate of 75.3%. Intubation failed in 33 (3.6%) patients, yielding an overall all success rate of 95.7% (*n* = 886).

### First pass success

In a multiple logistic regression model (R^2^ = 0.07; HL *p* = 0.96), adjusting for all variables in Table S1, an indication of ventilation was inversely associated with first pass success (OR = 0.42, 95% CI: 0.20–0.88, *p* = 0.02); deep sedation (OR = 0.56, 95% CI: 0.36–0.88, *p* = 0.13) and no drugs (OR = 0.47, 95% CI: 0.25–0.90, p = 0.02) compared to RSI was less likely to result in a first pass success; and increased on scene time (OR = 0.99, 95% CI: 0.985–0.997, *p* < 0.01) was inversely associated with first pass success.

### Overall success

In a multiple logistic regression model (R^2^ = 0.24; HL *p* = 0.46) adjusting for all variables in Table S1, deep sedation (OR = 0.17, 95% CI: 0.06–0.52, *p* < 0.01) and no drugs (OR = 0.24, 95% CI: 0.06–0.97, *p* = 0.04) compared to RSI was less likely to result in overall success.

### Failed intubation

In a multiple logistic regression model (R^2^ = 0.29; HL *p* = 0.76), adjusting for all variables in Table S1, deep sedation (OR = 8.87, 95% CI: 2.30–34.26, *p* < 0.01) and no drugs (OR = 9.71, 95% CI: 1.95–48.43, *p* < 0.01) compared to RSI was more likely to result in failed intubation. Increased on scene time was not associated with failed intubation (OR = 1.01, 95% CI: 0.999, 1.03, *p* = 0.079).

## Discussion

This study describes ETI in South Africa in terms of patient demographics, indications for intubation, means of intubation and success rates. To our knowledge, this is the largest study of paramedic-performed ETI from the African continent and other low-resource settings. We found that most patients who underwent ETI during this period were adults, males, trauma victims, or had a decreased level of consciousness following trauma. Non-physician ETI appeared to have high overall success rates, despite the presence of risk factors for difficult intubation. The most common approach to ETI was RSI.

South Africa, like many other LMICs, has a tremendously high trauma burden [22, 23]. It is therefore not surprising that the predominant reason for emergency care was following injury. Injury, and particularly TBI, is one of the most important contributors to morbidity and mortality in LMICs, especially in the younger, economically active population [24]. Out-of-hospital cardiac arrest was also a common presentation and this is likely reflective of an increasing burden of cardiovascular disease in Sub-Saharan Africa, including South Africa [25]. ETI in the setting of cardiac arrest is only recommended under optimal conditions and in settings with demonstrable high success rates, but has further been de-emphasised with chest compressions as the priority [26]. The utility of ETI in the South African context, where out-of-hospital cardiac arrest survival rates are very low [17, 27], is yet to be determined.

Across the world, non-physician ETI FPS rates range from 47 to 98% [28–30]. Further, a recent systematic review that was limited to ETI with an RSI approach only, found non-physician FPS of 78% (95% CI [65–89%]) [2]. The FPS rates reported in this study (75%) compares favourably to that reported in the international literature, despite comprising ETI approaches other than RSI. The use of neuromuscular blocking agents have been found to decrease the risk of difficult intubation [31], and yield a higher FPS rate [32]. This was also demonstrated in our study where RSI was shown to improve the odds of FPS over other approaches. Consequently, these other approaches could have had a lowering effect on the reported FPS. A third of cases had to be excluded because the number of intubation attempts was not recorded. It is not possible to know whether this was more likely to be noted when one or multiple intubation attempts were made. It is therefore conceivable that this could have influenced the reported FPS rate. Similarly, the overall success rates (95.7%) in this sample also compared favourably to that reported elsewhere (97% (95% CI [95 to 99%]) in RSI only [2]. When comparing these rates with non-physician ETI using multiple approaches, the overall success rate is slightly higher in our study than reported in a recent meta-analysis (91.7 (95% CI 61.6–100)) [3].

Owing to heterogeneity in prehospital emergency medical services across the world in terms of provider profile and skill level, and resourcing, comparisons of FPS is not always appropriate, and this should be taken into consideration when interpreting the results.

Perhaps then, it might be more appropriate to compare our FPS rates in this study with other studies originating from South Africa. A recent retrospective descriptive analysis of ETIs in Helicopter Emergency Medical Services (HEMS) reported a FPS rate of 79%, and an overall success rate of 98% [14]. This study included all approaches to ETI, and again compares favourably to the success rates reported herein for ground-based EMS. In another study, prehospital emergency care students achieved FPS and overall success rates of 85.2 and 92.4% when using an RSI approach only [13]. This is a higher FPS rate than reported in our study, but this could again be explained by the utility of neuromuscular blocking agents. Lastly, when comparing prehospital with emergency department success rates in South Africa, a recent study reported an FPS of 81.8%, which is considerably higher than reported herein. However, a sub-analysis of this sample reveals an FPS rate of 73.3% in cases where direct laryngoscopy was attempted, versus video laryngoscopy [33]. Video laryngoscopy was not available in the ground-based EMS involved in this study and thus, the latter FPS is a more appropriate comparison. Another consideration when comparing the FPS rates of this study with our results is the low proportion of trauma patients (20.9%). Manual in-line neck stabilisation, common practice during ETI in trauma victims, has been shown to significantly increase difficulty and failure of ETI [34].

Following multiple regression analysis, three factors remained associated with FPS: RSI approach, ventilation as an indication for intubation, and on scene time. RSI was associated with overall success, and inversely related to failed intubation. The impact of an RSI approach on intubation difficulty and success rates has already been discussed.

On scene time was inversely associated with FPS, while increasing on scene time was associated with overall failure. It is a logical conclusion that the requirement for multiple intubation attempts may prolong scene time, while successfully securing the airway on first attempt will limit the time spent on scene for stabilisation. This was demonstrated in a South African HEMS-based study where the number of clinical interventions were correlated with scene time, and every 1 additional intervention increased scene time by approximately 4 min [1]. Importantly though, interventions (with ETI being one of the most prevalent) did not result in a significantly more stable patient. The effect of prolonged scene time on mortality is yet to be determined in the South African context, especially with such a high burden of injury.

Ineffective ventilation as indication for ETI was inversely associated with FPS. This might be explained by the predictable instability associated with acidosis and hypoxaemia that accompany hypoventilation [35]. This may preclude prolonged attempts at securing the airway and result in earlier termination of an intubation attempt to avoid adverse events.

While success rates are a useful measure of airway management, they can be misleading as surrogates for safe ETI. Instead, there is a drive towards reporting of peri-intubation adverse events, rather than simply relying on success rates. In this retrospective study, it was difficult to validly extract AEs from the PRFs as the exact time of intubation was not recorded in most instances. Poor reporting was also the reason why a third of eligible cases where the number of intubation attempts were not recorded, had to be excluded. We therefore suggest that PRFs and/or reporting documents for all ETI instances be adjusted to allow for meaningful analysis as part of quality improvement and research practices. Using standardised airway forms has been found to reduce the rate of missing information and significantly increase the quality of data reported during prehospital ETI [36]. Calls for standardisation and robust clinical governance for prehospital intubation in South Africa, have been made previously [12] but there seem to be barriers to their implementation [37].

During the data collection period of this study, all advanced life support prehospital providers were licensed to intubate patients. In 2020, the Health Professions Council of South Africa (HPCSA) made the decision to remove ETI from the scope of practice of all prehospital providers and subsequently prehospital intubation can only be performed by degreed providers using the RSI approach. Currently, there are only 900 degreed providers registered with the HPCSA [38], yet it is unclear how many are actually still in full-time clinical practice in South Africa - a major concern as South Africa has had some considerable challenges in retention of prehospital providers [39, 40].

Emergency intubation in the prehospital environment is a complex intervention with severe complications if it is poorly planned or performed, so optimising all factors involved prior to intubation makes sense. This would include allowing only well-trained, competent individuals with adequate skills and experience to undertake ETI [41, 42]. While it can be seen as a commendable decision to reserve intubation for the highest qualified prehospital providers, this might translate into lack of access to a potentially life-saving intervention early in the course of emergency care. This may be undesirable, especially in TBI where early control of oxygenation and ventilation may prevent secondary brain injury [43] - TBI comprised over two-thirds of the patients in this study. The potential impact and unintended consequences of removing access to ETI in these patients warrants urgent examination. Some solutions to this may be to develop retention strategies for degreed paramedics and incentivise them to remain in practice, especially in rural or underserved communities. Another option may be regionalised scopes of practice for all ALS that are tailored to anticipated prehospital times and local injury and illness epidemiology.

Non-degreed paramedics are now licensed to insert supraglottic airway (SGA) devices as a primary means of securing the airway, while degreed paramedics often use SGAs as a rescue device in case of failed intubation. In other settings, SGAs have been shown to be safe and effective means for securing the airway and achieving oxygenation and ventilation in the prehospital setting [44]. This is especially true in cardiac arrest [45], a major indication for ETI in this study. However, their role in trauma is less clear with limited data [44]. There are currently no studies on the use of SGA as primary airway device from the South African setting and this should be considered in future however, its use as primary device shows some promise.

It is essential to acknowledge the paucity of robust data on the effect of prehospital ETI on morbidity and mortality [46], especially in trauma [6, 42, 47]. Where data exists, it is mostly from high income settings, or cannot allow for meaningful comparison or meta-analysis owing to health system variation, selective reporting, or risk of bias. There is an urgent need to perform additional research that evaluates the peri-intubation safety and outcome following prehospital ETI. In our view, the only way that this could happen robustly is through the implementation of mandatory standard reporting databases.

### Limitations

This retrospective study is not without limitations. Data were extracted from self-reported clinical notes that are not intended for research and therefore had to be extracted based on the clinical impression of the extractors. Certain data such as airway difficulty and aggravating factors relating to airway assessment are based on the subjective judgement of the practitioner performing the assessment and can therefore only be considered as estimates of potential airway difficulty. Only instances of prehospital ETI were included in this study and other methods of basic or advanced airway management was not studied. External validity is certainly affected by the inclusion of private services and only one, provincial public emergency medical service. Other smaller, local private EMS were also excluded. External validity is also influenced by the relatively well-developed prehospital system in South Africa, as compared to other LMICs. This limits the immediate generalisability to other countries in Africa however, the results here may be of interest in settings where the EMS system is just developing. Another important limitation is that no patients that did not have ETI were included to allow for comparisons of outcome and scene time delays associated with ETI.

## Conclusion

In this sample of ground-based EMS non-physician ETI, we found success rates similar to that reported in international literature on non-physician ETI. Success rates also compared favourably to South African facility-based rates, when intubation is performed by physicians. RSI, on scene time and ineffective ventilation as an indication for intubation were the most important variables associated with FPS. More research is needed to determine AE rates and the impact of prehospital ETI on patient outcome. There is an urgent need to standardise airway management reporting in South Africa.

## Supplementary Information

**Additional file 1.**

## Data Availability

The datasets used and analysed during the current study are available form the corresponding author on reasonable request.

## Abbreviations

A/W: Airway
AEs: Adverse events
ALS: Advanced Life Support
CPR: Cardiopulmonary Resuscitation
EMS: Emergency Medical Services
ePRF: Electronic Patient Report Form
ETI: Endotracheal Intubation
FPS: First Pass Success
HEMS: Helicopter Emergency Medical Services
HIC: High Income Country
HL: Hosmer-Lemeshow goodness of fit test
HPCSA: Health Professions Council of South Africa
LMIC: Low- to Middle-income Country
LOC: Level of Consciousness
MILNS: Manual In-line Neck Stabilisation
PRF: Patient Report Form
RSI: Rapid Sequence Intubation
SGA: Supraglottic Airway
TBI: Traumatic Brain Injury
TMD: Thyromental Distance
VIF: Variance Inflation Factor
