# Supplementary material for: A retrospective descriptive analysis of non-physician-performed prehospital endotracheal intubation practices and performance in South Africa
Source: BMC Emerg Med. 2022 Jul 16;22:129. doi: 10.1186/s12873-022-00688-4 (PMC9287876; doi:10.1186/s12873-022-00688-4)
Supplement: Supplementary file 1 — Additional file 1. [file 12873_2022_688_MOESM1_ESM.docx]

| Table S1 |  |  |  |  |
| --- | --- | --- | --- | --- |
|  |  | First Pass Success | Overall success | Failed completely^a^ |
| Variables | All available data (n = 926) | Complete cases (n = 793) | Complete Cases (n = 793) | Complete Cases (n = 793) |
|  | n (%) | Odds ratios (95%CI) | Odds ratios (95%CI) | Odds ratios (95%CI) |
| Age |  |  |  |  |
| Paediatric | 79 (8.53) | Reference | Reference | Reference |
| Adult | 781 (84.34) | 1.50 (.84, 2.67) | 0.60 (.13, 3.5) | 0.99 (.16, 6.29) |
| Gender |  |  |  |  |
| Male | 533 (57.56) | Reference | Reference | Reference |
| Female | 371 (40.06) | 1.19 (.81, 1.745) | 0.75 (.31, 1.83) | 1.16 (.42, 3.17) |
| Reason for intubation |  |  |  |  |
| blunt | 126 (13.61) | Reference | Reference | Reference |
| penetrating | 25 (2.7) | 6.72 (.78, 58.02) | 2.63 (.22, 30.82) | empty |
| tbi | 328 (35.42) | 0.83 (.44, 1.58) | 2.67 (.57, 12.42) | 0.83 (.12, 5.60) |
| other trauma | 23 (2.48) | 3.56 (.70, 18.09) | empty | empty |
| cardiac arrest | 204 (22.03) | 0.60 (.22, 1.68) | 0.94 (.09, 9.46) | 1.88 (.07, 49.99) |
| resp | 58 (6.26) | 0.97 (.40, 2.36) | 1.25 (.23, 6.96) | 1.81 (.25, 12.87) |
| tox | 62 (6.7) | 0.79 (.33, 1.91) | 0.64 (.14, 2.96) | 3.50 (.58, 21.06) |
| sepsis | 1 (0.11) | empty | empty | empty |
| other medical | 13 (1.40) | 0.89 (.16, 4.93) | empty | empty |
| stroke | 54 (5.83) | 0.62 (.26, 1.43) | 0.67 (.16, 2.85) | 2.88 (.53, 15.79) |
| other neuro | 30 (3.24) | 1.49 (.47, 4.73) | empty | empty |
| psych | 1 (0.11) | empty | empty | empty |
| Indication for intubation |  |  |  |  |
| GCS | 515 (55.62) | 0.83 (.41, 1.72) | 0.47 (.13, 1.68) | 1.73 (.40, 7.50) |
| Hypoxia | 11 (1.19) | 0.42 (.08, 2.09) | 0.16 (.01, 3.82) | omitted |
| Ventilation | 96 (10.37) | **0.42 (.20, .88)** | 0.72 (.20, 2.60) | 1.10 (.26, 4.67) |
| ExistingAO | 49 (5.29) | 0.70 (.28, 1.75) | 0.28 (.06, 1.23) | 3.81 (.69, 21.17) |
| ImpendingAO | 45 (4.86) | 1.56 (.50, 4.87) | 0.40 (.08, 2.06) | 1.52 (.23, 9.96) |
| Combative | 48 (5.18) | 1.55 (.49, 4.93) | 0.41 (.04, 4.85) | 3.57 (.23, 55.83) |
| Humanitarian | 7 (0.76) | omitted | omitted | omitted |
| Cardiac arrest | 249 (26.89) | excluded (vif) | excluded (vif) | excluded (vif) |
| Failure of airway device | 1 (0.11) | omitted | omitted | omitted |
| Approach |  |  |  |  |
| RSI | 344 (37.15) | Reference | Reference | Reference |
| Deep sedation | 256 (27.65) | **0.56 (.36, .88)** | **0.17 (.06, .52)** | **8.87 (2.30, 34.26)** |
| No drugs | 89 (9.61) | **0.47 (.25, .90)** | **0.24 (.06, .97)** | **9.71 (1.95, 48.43)** |
| CPR | 236 (25.49) | 0.82 (.29, 2.30) | 0.20 (.02, 2.22) | 3.78 (.13, 109.44) |
| Risk factors not assessed | 342 (36.93) | 0.99 (.47, 2.08) | 1.17 (.17, 8.17) | 0.33 (.03, 3.75) |
| Risk factors |  |  |  |  |
| No risk factors | 68 (11.64) | 0.94 (.36, 2.47) | 1.51 (.17, 13.33) | 0.47 (.04, 6.36) |
| Prior difficulty intubation | 8 (1.37) | 0.50 (.09, 2.87) | 0.84 (.05, 13.16) | 0.52 (.03, 10.46) |
| Reduced neck mobility | 363 (62.16) | 0.68 (.29, 1.58) | 1.46 (.20, 10.53) | 0.37 (.03, 4.84) |
| Severe obesity short neck | 20 (3.42) | 0.34 (.11, 1.03) | 0.51 (.07, 3.87) | 1.57 (.15, 16.52) |
| Limited mouth opening | 12 (2.05) | 0.27 (.06, 1.20) | 0.64 (.04, 10.40) | 0.95 (.04, 24.10) |
| Short TMD | 3 (0.51) | 0.65 (.04, 11.15) | omitted | omitted |
| Trauma | 72 (12.33) | 0.88 (.42, 1.82) | 1.11 (.14, 9.05) | 1.07 (.08, 14.01) |
| Secretions blood mucous | 205 (35.10) | 0.58 (.33, 1.02) | 3.10 (.54, 17.68) | 0.20 (.02, 1.84) |
| Ariway device not working | 9 (1.54) | 0.26 (.05, 1.45) | 0.05 (.003, 1.04) | 12.96 (.46, 368.63) |
| Aggrevating conditions not assessed | 194 (20.95) | 1.08 (.27, 4.43) | 0.06 (.003, 1.04) | 5.96 (.08, 472.70) |
| Aggrevating conditions |  |  |  |  |
| No aggrevating conditions | 479 (65.44) | 1.24 (.32, 4.91) | 0.38 (.02, 8.17) | .63 (.01, 60.57) |
| Entrapped | 45 (6.15) | 1.87 (.49, 7.06) | 0.11 (.007, 1.69) | omitted |
| Access | 15 (2.05) | 4.31 (.53, 35.39) | 0.30 (.04, 2.93) | 1.94 (.07, 56.52) |
| Positioning | 6 (0.82) | 0.52 (.06, 4.98) | 0.70 (0.04, 13.997) | omitted |
| Light | 12 (1.64) | 1.47 (.23, 9.29) | omitted | omitted |
| Darkness | 96 (13.11) | 1.34 (.34, 5.33) | 0.81 (.03, 22.34) | omitted |
| Hostile | 42 (5.74) | 2.54 (.72, 8.96) | 0.16 (.01, 2.03) | 2.78 (.03, 260.79) |
| Moving vehicle | 29 (3.96) | 0.67 (.14, 3.30) | 0.16 (.006, 4.31) | omitted |
| Stationary vehicle | 57 (7.79) | 1.09 (.26, 4.59) | 0.05 (.003, .83) | 15.43 (.21, 1128.06) |
| Mean Scenetime (SD; n) | 45.7 (28.33; 871) | **0.99 (.985, .997)** | 0.995 (.98, 1.01) | 1.01 (.999, 1.03) |
| Number of observations |  | 784 | 710 | 572 |
| McFadden's R2 |  | 0.074 | 0.239 | 0.292 |
| AUC |  | 0.69 | 0.86 | 0.90 |
| Correctly classified |  | 76.28% | 94.79% | 95.10% |
| Hosmer-Lemeshow |  | p = .964 | p = .456 | p = .762 |
| a. estimate with one case excluded due to large values on diagnostics | | | | |
